# Construction of a Modular Arsenic-Resistance Operon in *E. coli* and the Production of Arsenic Nanoparticles

**DOI:** 10.3389/fbioe.2015.00160

**Published:** 2015-10-20

**Authors:** Matthew Charles Edmundson, Louise Horsfall

**Affiliations:** ^1^Institute of Cell Biology, School of Biological Sciences, University of Edinburgh, Edinburgh, UK

**Keywords:** biogenic nanoparticles, arsenic nanoparticles, arsenic decontamination, arsenic reduction, modular arsenic-resistance construct

## Abstract

Arsenic is a widespread contaminant of both land and water around the world. Current methods of decontamination such as phytoremediation and chemical adsorbents can be resource and time intensive, and may not be suitable for some areas such as remote communities where cost and transportation are major issues. Bacterial decontamination, with strict controls preventing environmental release, may offer a cost-effective alternative or provide a financial incentive when used in combination with other remediation techniques. In this study, we have produced *Escherichia coli* strains containing arsenic-resistance genes from a number of sources, overexpressing them and testing their effects on arsenic resistance. While the lab *E. coli* strain JM109 (the “wild-type”) is resistant up to 20 mM sodium arsenate, the strain containing our plasmid pEC20 is resistant up to 80 mM. When combined with our construct pArsRBCC arsenic-­containing nanoparticles were observed at the cell surface; the elements of pEC20 and pArsRBCC were therefore combined in a modular construct, pArs, in order to evaluate the roles and synergistic effects of the components of the original plasmids in arsenic resistance and nanoparticle formation. We have also investigated introducing the lac operator in order to more tightly control expression from pArs. We demonstrate that our strains are able to reduce toxic forms of arsenic into stable, insoluble metallic As(0), providing one way to remove arsenate contamination, and which may also be of benefit for other heavy metals.

## Introduction

Arsenic contamination is a major problem globally, both from human and geological sources. High levels of arsenic are found in the soil of many areas in countries such as the UK and China (van Elteren et al., 2006; Chen et al., 2015) and in a number of countries, including Argentina, India, and Mexico, geological contamination of agricultural land by arsenic is exacerbated by the use of arsenic-contaminated water in irrigation (Rosas-Castor et al., 2014). Arsenic is also a major contaminant of ground water used for human consumption in countries such as Bangladesh and Nepal (Singh et al., 2015). Synthetic biology is already being used to develop biosensors to detect arsenic contamination in water supplies, utilizing modular DNA components to create sensitive whole-cell systems that can be used in areas where traditional detection methods are not suitable (Joshi et al., 2009; de Mora et al., 2011). This system, utilizing *Bacillus subtlis* as a chassis, is currently being developed for commercialization^1^. However, while this is a valuable way to identify arsenic contamination, it does not address how to remove the arsenic.

There are a number of methods employed or under consideration that aim to remove arsenic contamination. These include the use of plants for phytoremediation, e.g., *Pteris vitatta* (Sugawara et al., 2014), to hyper-accumulate arsenic and remove it from soil, and the use of granular titanium dioxide to adsorb arsenic from contaminated water (Bang et al., 2005). However, these methods have drawbacks. For instance, phytoremediation requires a long-term commitment; the plants used require time to grow, several harvests, and re-plantings may be required; additionally, issues surround the introduction of non-native plants. There is also arsenic-contaminated plant biomass produced which must then be disposed of; this is usually done by incineration, with the potential for arsenic release into the air, and ashes taken to designated hazardous waste dumps (Ali et al., 2013). Using adsorbent materials is also not without problems as they must be replaced or recharged regularly (German et al., 2014), and there may be transportation difficulties for remote areas.

Arsenic decontamination using bacteria offers a way to circumvent these problems. In contrast to materials such as TiO_2_, bacteria are self-renewing and do not need to be re-charged thus transport costs are lower as only a small initial inoculant is required to begin the process. Additionally, bacteria are capable of not only absorbing arsenic contaminants but also converting it to other, less harmful forms (Kao et al., 2013). To improve traditional phytoremediation, it could be coupled to bacterial decontamination, where the contaminated plant material can be used as a feedstock for the bacteria, converting the arsenic to less toxic forms and preventing the release of arsenic by burning.

When arsenic is found as a contaminant it is usually present in either the form of arsenite [As(III)] or arsenate [As(V)] (Smedley and Kinniburgh, 2002). Since As(III) is the most detrimental to human health (Hughes, 2002), it would seem a good strategy to engineer an organism able to oxidize As(III) to the “safer” As(V), and indeed a number of efforts have focused on oxidation (Kao et al., 2013). However, As(V) is sensitive to pH changes (Smedley and Kinniburgh, 2002), so it is possible for a formerly “de-contaminated” site to become unsafe again as As(V) reverts to As(III). In addition, As(V) itself is toxic as it can be accidentally incorporated in place of phosphate by biological systems (Saltikov and Olson, 2002), inhibiting glycolysis and phosphorylation (Sung et al., 2009). Therefore converting arsenic into its elemental, insoluble As(0) form may be the safest route to detoxification, despite the requirement to reduce As(V) to As(III) as a necessary step to achieving As(0).

In order to do this, we engineered a bacterium to remove arsenic using a synthetic biology approach to create a modular genetic construct containing genes involved in arsenic resistance. The effects of each module were investigated with the removal of parts as required. To this end, we have investigated a number of arsenic-resistance genes from the anaerobic bacterium *Desulfovibrio alaskensis* strain G20 (Li and Krumholz, 2007), both singly and in combination, by transforming them into *E. coli* cells. The *D. alaskensis* genes are the arsenate reductases ArsC1, ArsC2 and ArsC3, the arsenic efflux pump ArsB, and the Ars operon repressor protein ArsR (used in conjunction with the repressible Ars operon promoter).

We have also included the modular use of a phytochelatin analog, EC20 (Bae et al., 2002) ([Glu-Cyt]_20_-Gly). Phytochelatins are metal-chelating peptides, produced enzymatically in plants, which are capable of binding to a number of heavy metals and metalloids, including arsenic (Zhang et al., 2012). Phytochelatin synthetase has been successfully expressed in *E. coli*, allowing the cells to produce nanoparticles of a number of different metals, including CdZn, CdSe, CdTe, and SeZn (Park et al., 2010). However, we chose to use EC20, a synthetic phytochelatin, directly translated from mRNA rather than made enzymatically. It has previously been expressed in bacteria to chelate a number of heavy metals, including cadmium (Bae et al., 2002), mercury (Bae et al., 2001, 2002), zinc, lead, copper, nickel, and molybdenum (Biondo et al., 2012). Encoding EC20 in this way, and not using an enzyme to produce it, allowed us to localize it to the outer membrane of the cell by attaching EC20 to the membrane-spanning domain of IgA protease from *Neisseria gonorrhoeae* (referred to as EC20/IgA). We have tested these components both separately and as part of a unified, modular arsenic-resistance/removal plasmid. We have demonstrated that our engineered *E. coli* is able to not only tolerate high levels of arsenic, but is also able to convert the arsenic to an insoluble elemental form, removing large amounts of arsenic from solution.

## Materials and Methods

### Strains and Plasmids

The vector pArsC1 is based on the pUC19 plasmid; pUC19 and pArsC1 confer ampicillin resistance. pEC20 is based on pHEβ, ultimately derived from the pAK100 vector (Krebber et al., 1997); pHEβ and pEC20 confer chloramphenicol resistance. pArsRBCC is based on pUC57-Kan; pUC57-Kan and pArsRBCC confer kanamycin resistance. The pET28b vector was also used as a kanamycin resistance-conferring control plasmid. The modular constructs pArs and pArs_lac both have pUC57-Kan as the vector backbone and confer kanamycin resistance (plasmid origins shown in Table S1 in Supplementary Material).

All genes in pArsC1, pArsRBCC and pArs have been ameliorated for expression in *E. coli*; these vectors were synthesized by Genewiz Inc., NJ, USA. The sequences of the ameliorated genes are shown in Figure S1 in Supplementary Material. pHEβ containing EC20/IgA was kindly supplied by Dr. Luis Angel Fernández Herrero, Centro Nacional de Biotecnologia, Madrid. Protein expression of all strains is shown in Figure S2 in Supplementary Material.

### pArs_lac Creation

pArs_lac was created using pArs as a template by using primers containing a pArs-specific sequence and part of the lac operator sequence. The primers used (“Lac operon forward” and “Lac operon reverse,” Figure S3 in Supplementary Material) were designed to be back-to-back, thereby producing full-length linear pArs plasmid with the lac operator sequence overhanging, half at the 3′ and half at the 5′ end. PCR conditions: initial denaturation 98°C, 30 s; 35 cycles of 98°C, 30 s, 72°C, 3 min 30 s; final elongation 72°C, 10 min. The primers were phosphorylated at the 5′ end so that after PCR, the linear vector could be circularized using T4 DNA ligase, and then transformed into *E. coli* TOP10 chemically competent cells (ThermoFisher), and subsequently transferred to *E. coli* JM109 cells.

### *E. coli* Resistance to Arsenic on As-Containing Agar Plates

All experiments were performed using the laboratory *E. coli* strain JM109. All *E. coli* cultures were grown for 16 h (37°C, 200 rpm) in LB medium supplemented with the appropriate antibiotic (final concentration of 50 μg/ml ampicillin for pUC19 and pArsC1; 40 μg/ml for pHEβ and pEC20; 50 μg/ml kanamycin for pET28b, pArsRBCC, pArs, and pArs_lac). All cultures were equalized to an OD_600_ of 0.65–0.75. Serial dilutions to 1 in 100, 1 in 10,000 and 1 in 1,000,000 performed. 10 μl of equalized culture or dilutions thereof were spotted onto LB agar plates containing appropriate antibiotic(s) and varying concentrations of sodium arsenate and/or 0.1 mM IPTG. Plates were incubated at 37°C for 16 h, followed by a 3 days of incubation at 25°C.

### *E. coli* Incubation with Arsenic in MOPS pH 7.5 Buffer

Cultures were grown for 16 h (37°C, 200 rpm) in LB medium. Cells were isolated by centrifugation at 3200 *g* for 10 min, and re-suspended in 10 mM MOPS buffer, pH 7.5. For cultures <10 ml, the volume of MOPS used was equal to the original culture volume; for larger cultures one tenth the volume of the original culture was used. This wash step was performed three times before the resulting pellets were again re-suspended in the appropriate volume of MOPS buffer. Arsenic was added to the samples in the form of sodium arsenate to a final concentration of either 2 or 5 mM. The samples were incubated at 25°C for 7 days, or at 25°C for 3 days followed by 4°C for 4 days, depending on the experiment.

### Transmission Electron Microscopy Imaging

Cells, incubated in MOPS buffer with arsenic as described above, were drop-cast onto a 200-mesh copper grid and incubated at 25°C for 10–20 min, excess liquid removed by blotting paper and the grid air-dried. The transmission electron microscope was a Philips CM120, with images taken on a Gatan Orius CCD camera.

### Inductively Coupled Plasma Optical Emission Spectrometry

*E. coli* cells of the appropriate strains were incubated in MOPS buffer with arsenic as described above. The cells were removed by centrifugation at 3200 × *g* for 10 min and the supernatants filtered using a 0.22 μm filter before ultracentrifugation at 270,000 × g for 1.5 h. Supernatants were analyzed by inductively coupled plasma optical emission spectrometry (ICP–OES) using a Perkin Elmer Optima 5300 DV at the School of Chemistry, University of Edinburgh.

### Energy Dispersive X-ray Spectroscopy

Samples were prepared following the protocol for TEM imaging as described above. The TEM used for energy dispersive X-ray spectroscopy (EDX) was a JEOL ARM 200 Cold FEG TEM operated at 200 kV accelerating voltage in the scanning transmission mode with Cs aberration corrected STEM. The conditions used for the collection of the X-ray spectra were: convergence angle of the electron probe 29 mrad with a probe current of approximately 0.2 nA. The spectra were collected using a Bruker XFlash5060 SDD spectrometer controlled *via* Gatan Digital Micrograph software configured for fast spectrum imaging (1000 spectra/s).

## Results

### Arsenic Resistance

**Figure 1:**
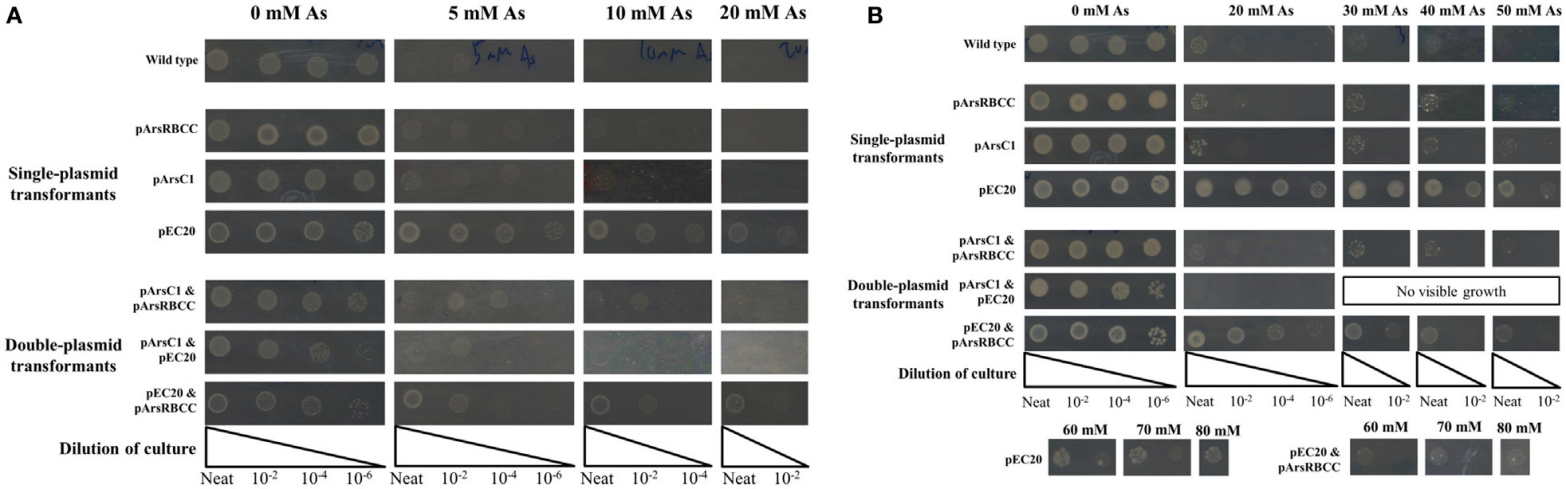
***E. coli* singly and doubly transformed strains gown on agar plates containing varying concentrations of sodium arsenate after (A) 16 h and (B) 3 days of incubation**.

The JM109 laboratory strain of *E. coli* displays a non-trivial level of arsenic resistance, showing growth but reduced colony density after 16 h in the presence of 5–10 mM sodium arsenate when compared to *E. coli* grown in the absence of arsenic; after 3 days incubation JM109 grows relatively well in the presence of up to 20 mM sodium arsenate and growth is completely abolished by the addition of 40 mM sodium arsenate (Figures 1A,B). This can be attributed to *E. coli*’s native arsenic resistance mechanism encoded on an operon and containing the genes *arsRDABC* (Carlin et al., 1995). To supplement this natural arsenic resistance three plasmids were introduced into *E. coli* cells, each carrying additional arsenic-resistance genes, namely pArsC1, pEC20, and pArsRBCC. pArsC1 and pArsRBCC encode arsenic-resistance genes from the anaerobic bacterium *Desulfovibrio alaskensis*; pArsC1 contains the arsenate reductase *arsC1* (accession no. WP_011368621)^2^; pArsRBCC contains two arsenic reductases, *arsC2* (accession no. ABB395882) and *arsC3* (accession no. ABB39589), the arsenite efflux pump *arsB* (accession no. Q30XK9), and the repressor *arsR* (accession no. AEL79451), which controls the arsenic-inducible promoter that all four genes are transcribed from. pEC20 is built around the phytochelatin analog peptide EC20, which is a synthetic phytochelatin as in nature phytochelatins are synthesized enzymatically but here we encode it directly in the DNA sequence. The DNA sequence of EC20 is immediately followed by that of the membrane domain of IgA protease from *N. gonorrhoeae* (accession no. WP_047917625), so that they are expressed together forming the hybrid protein EC20/IgA. EC20/IgA also carries a signal sequence for localization to the outer membrane.

**Figure 2:**
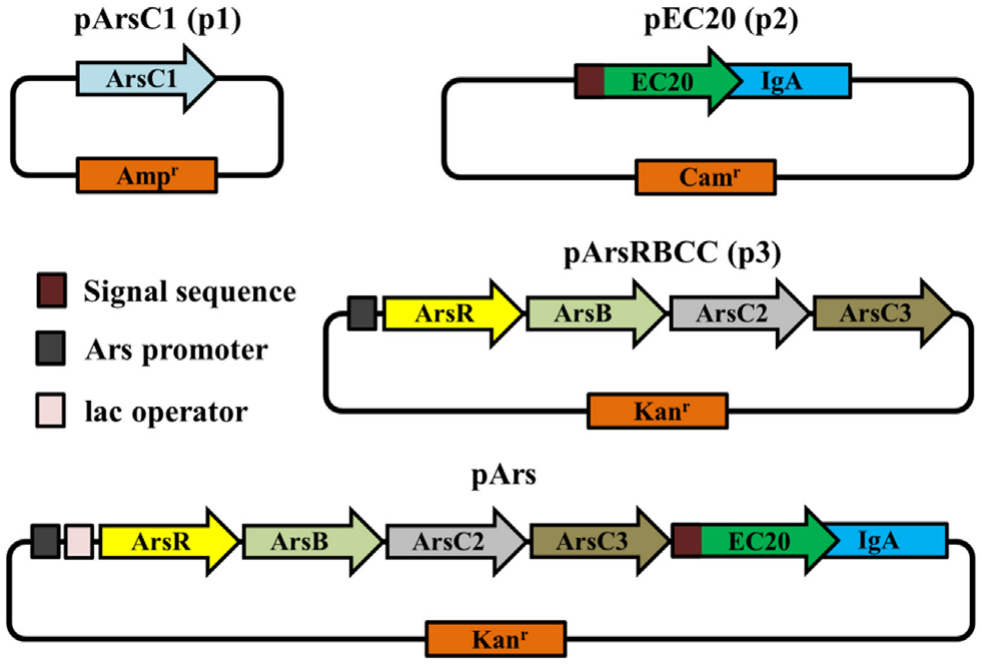
**Schematic representations of vectors used in this study**.

When grown on media supplemented with arsenic, pArsC1 and pArsRBCC have a negligible impact on *E. coli* growth after 16 h incubation, with both showing similar growth to the control cells (Figure 1A). However, after 3 days, both strains performed somewhat better than the control cells, showing growth at 50 mM sodium arsenate (Figure 1B). Cells containing pEC20 showed the highest level of arsenic resistance; after 16 h incubation, there was still significant growth at 30 mM sodium arsenate, while after 3 days, there was no difference between the colony density at 0 and 30 mM sodium arsenate, and significant growth in the presence of 80 mM sodium arsenate was evident (Figures 1A,B). The synergistic effects of all three combinations of plasmid pairings in *E. coli*, namely pArsC1 with pEC20, p1 + 2; pArsC1 with pArsRBCC, p1 + 3; and pEC20 with pArsRBCC, p2 + 3 (Figure 2), were investigated. p1 + 2 and p1 + 3 performed roughly on par with the control cells after 16 h incubation, and after 3 days showed slightly higher resistance than the control cells but neither was as resistant as the strains containing just a single plasmid (Figures 1A,B). p2 + 3, however, achieved a high level of synergy, as after 16 h incubation there was significant growth at 20 mM sodium arsenate, while after 3 days of incubation, there was a high colony density observed at 50 mM sodium arsenate, and visible growth in the presence of 80 mM sodium arsenate.

### Arsenic Nano-Structures

**Figure 3:**
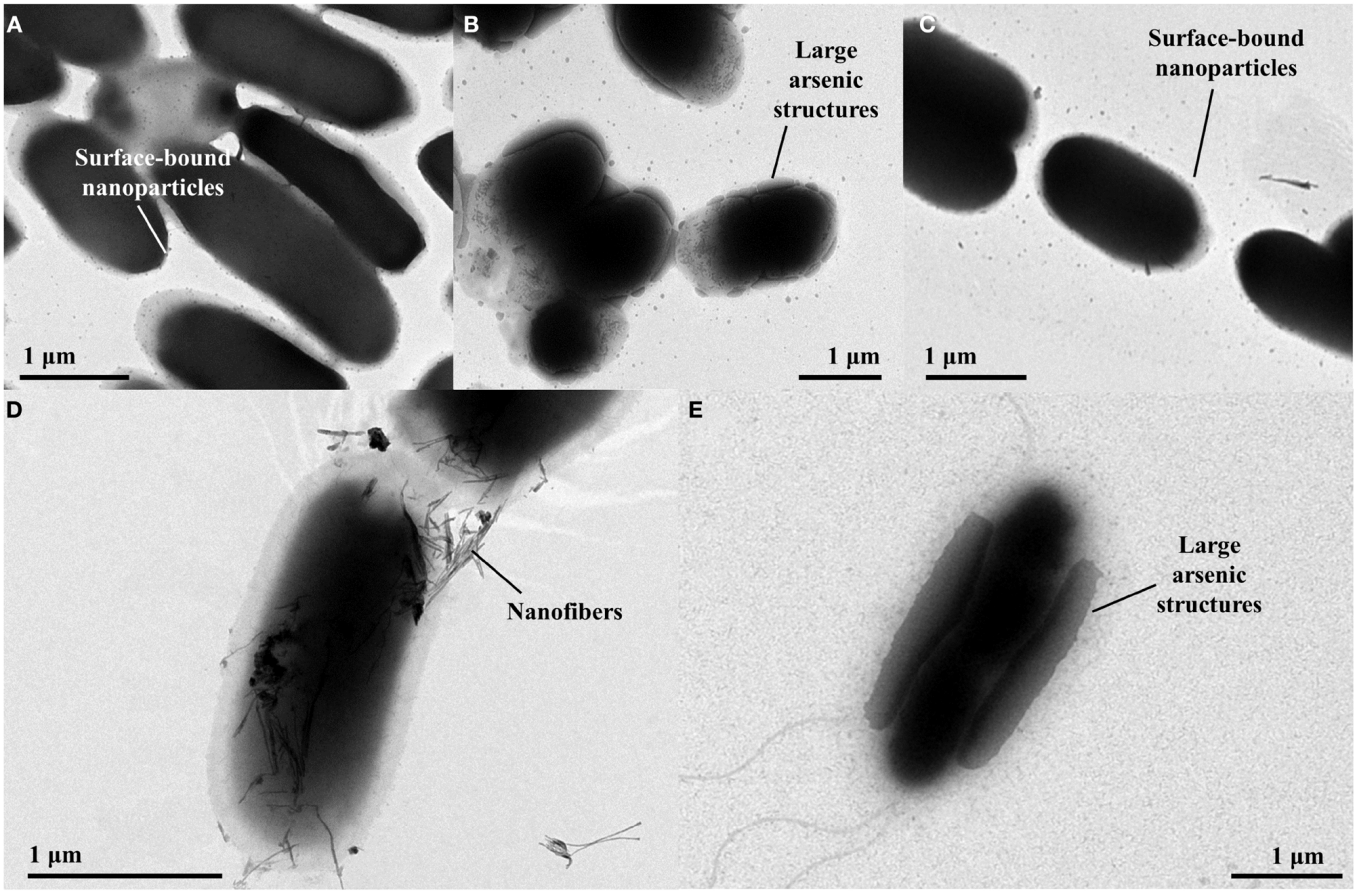
**TEM images of E. coli strains containing different vector combinations incubated in MOPS buffer containing sodium arsenate. (A)** pHEβ (WT) cells in 2 mM sodium arsenate with small particles attached to the cell surface; **(B)** pEC20 & pArsRBCC (p2 + 3) cells in 2 mM sodium arsenate with large structures attached to the cell surface; **(C)** pEC20 cells in 2 mM sodium arsenate with small particles attached to the cell surface; **(D)** pEC20 & pArsRBCC (p2 + 3) cells in 2 mM sodium arsenate with nanofibers; **(E)** pEC20 & pArsRBCC (p2 + 3) cells in 5 mM sodium arsenate with very large structures attached to the cell surface.

Transmission Electron Microscopy imaging of control cells containing the plasmid pHEβ incubated in 2 mM sodium arsenate revealed that the cells accumulate electron-dense structures at the cell surface that are 20–30 nm in diameter (Figure 3A). Cells containing p2 + 3 incubated under the same conditions produced much larger electron-dense structures at their cell surface, some being almost 1 μm in length (Figure 3B); however when only p2 is present the size of the arsenic structures is small, comparable to the control cells (Figure 3C), suggesting that the arsenate reductases encoded on p3 are required for the production of the larger structures. For the p2 + 3 strain, in addition to the arsenic nanoparticles there are also fibrous structures present (Figure 3D). These nanofibers are around 5 nm across and up to 0.5 μm in length, and at high magnification it can be seen that electron-dense structures 2–3 nm in diameter are attached to them. Cells containing only pEC20 do not produce these nanofibers.

Increasing the arsenic concentration from 2 to 5 mM effects the size of the surface-bound structures in both control cells and p2 + 3 cells. Again, cells containing the arsenic resistance plasmids produce larger structures and these cover a large area of the cell surface (Figure 3E). At higher magnification, large amounts of small fibers are observed for this strain. Increasing the initial arsenic concentration, therefore, influences nanoparticle morphology, increasing the size of the cell surface-bound structures while decreasing the length of the nanofibers.

**Figure 4:**
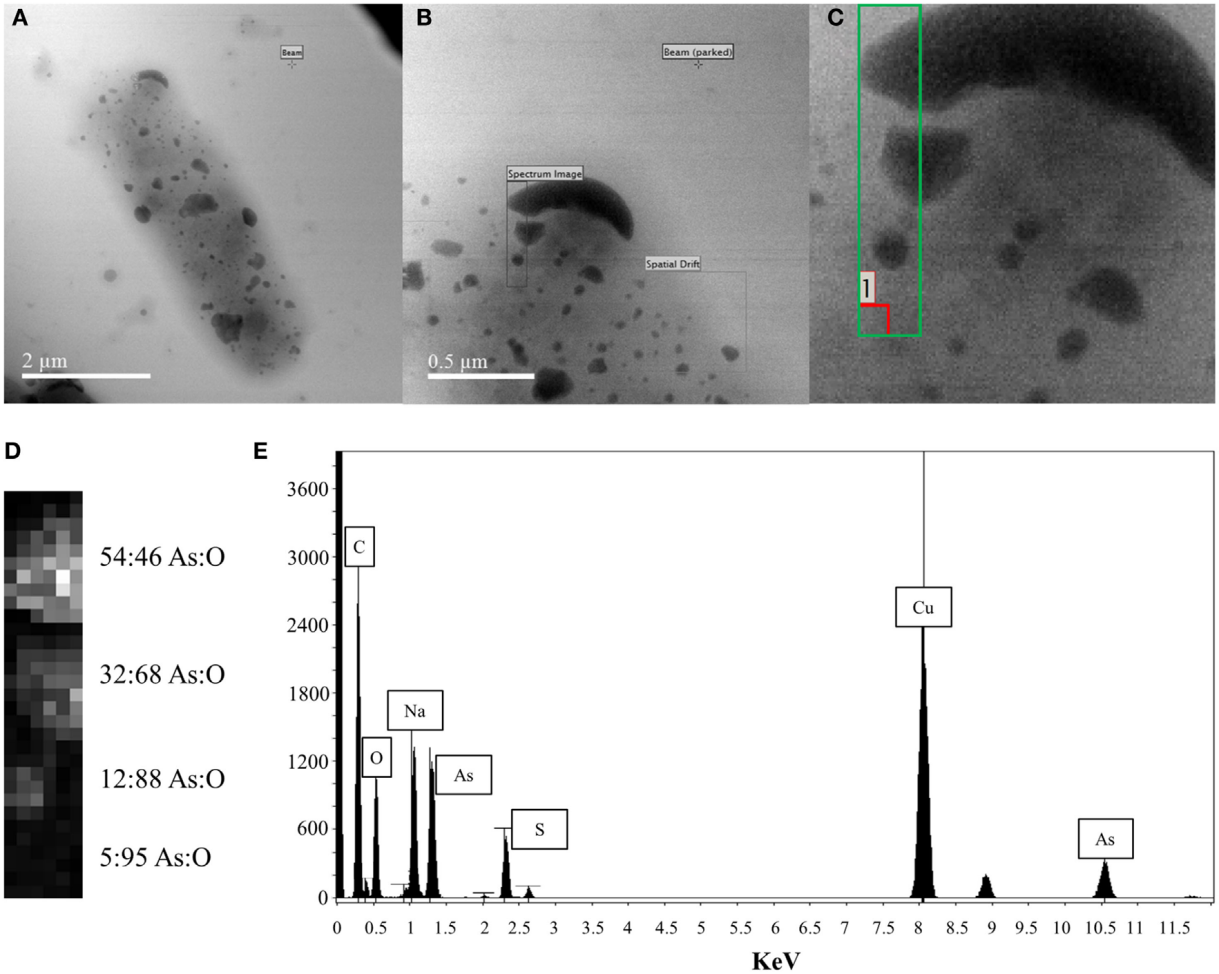
**EDX analysis of large cell surface-bound arsenic structures produced by pEC20 & pArsRBCC (p2 + 3) cells (A) TEM image of cell with bound arsenic; (B) close-up of analyzed area; (C) green box indicates where beam was placed, red box indicates where background reading was taken; (D) atomic% from X-ray spectra, last reading (5:95 As:O) indicates background reading of support material; (E) summed X-ray spectrum from the analyzed region showing all elements present**.

Energy Dispersive X-ray Spectroscopy analysis was performed on the surface-bound structures on the p2 + 3 cell depicted in the scanning electron micrograph in Figure 4A. The area investigated is shown by a black rectangle in Figure 4B, with a close-up of the area in the green box in Figure 4C and a contrast image of this area in Figure 4D. The contrast of the large structures against the background is apparent, with the high-density regions showing the highest proportion of arsenic present with a 54:46% As:O ratio; in the less dense areas more oxygen is seen, indicating a large amount of oxygen is associated with the support material, with an As:O ratio of 5:95%. Taking this high-oxygen background into account, along with the overall composition of this area (Figure 4E), the most likely composition of the large surface-bound structures is primarily metallic As(0), with an oxidized outer layer.

### Modular Construct

**Figure 5:**
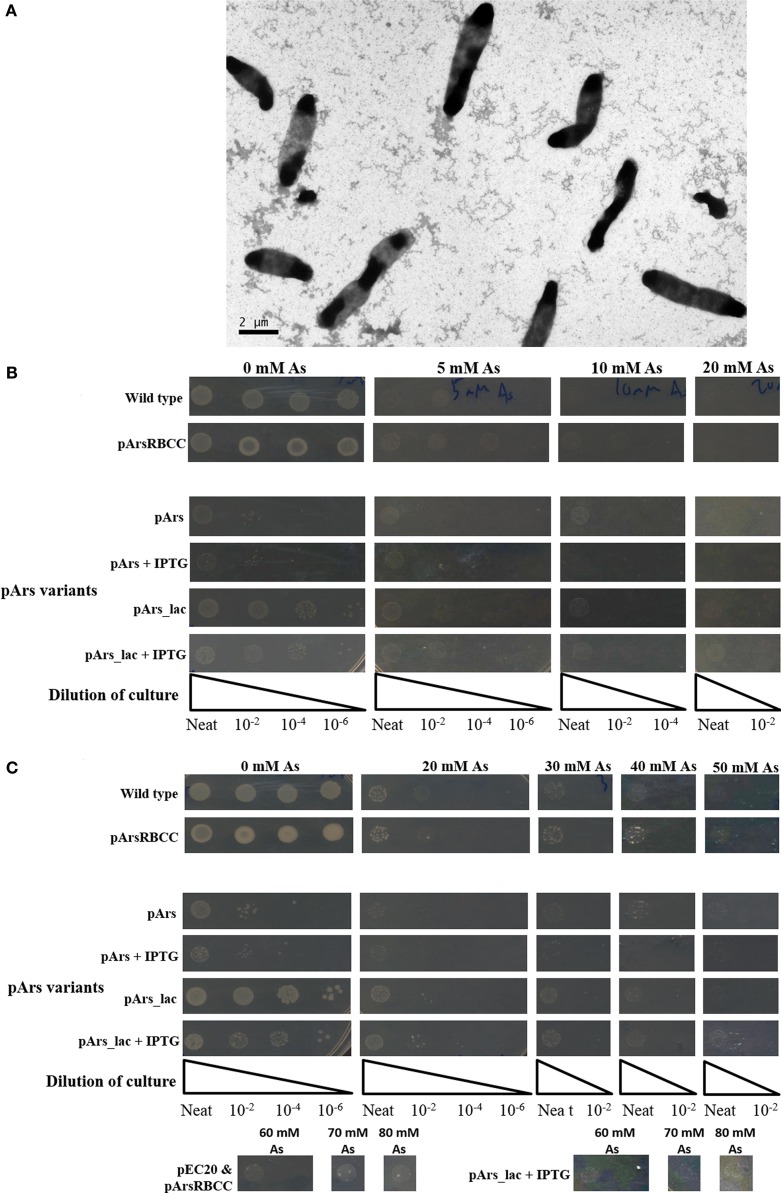
**(A)** Cells containing pArs after 16 h incubation at 37°C; **(B)** comparison of WT cells with pArs and pArs_lac cells, with and without IPTG induction, at various arsenic concentrations after 16 h incubation at 37°C and **(C)** after 3 days of incubation at 25°C.

While the outer membrane located phytochelatin EC20 encoded on the plasmid pEC20 confers a high degree of arsenic resistance to cells, nanofiber formation is observed only when it is paired with pArsRBCC. Therefore we decided to combine these useful genetic elements into a single construct using DNA synthesis to codon-optimize genes for expression in *E. coli* (Figure 2), producing the plasmid pArs. In addition, the *E. coli* ArsR (accession no. WP_000008958) and Ars operon promoter were used in place of the *D. alaskensis* sequences, and a number of unique restriction sites were inserted to allow each individual gene to be replaced or removed in a modular fashion. However, this new construct had a detrimental effect on growth (Figure 5A). OD_600_ measurements overestimated the number of colony forming units (CFUs) present, and even in the absence of arsenic cells were not dividing correctly. This was corrected through a tightening of operon expression by the addition of the lac operator, which was inserted into the Ars promoter downstream of the ArsR binding site.

Use of the lac operator immediately improved the growth rate in liquid culture of cells containing pArs_lac compared to pArs, with pArs_lac showing more CFUs than pArs (Figure 5B). On plates containing 0.1 mM IPTG the pArs_lac colonies are smaller than those of the control cells and the colony density slightly reduced, although pArs_lac cells still outperform pArs cells (Figure 5B). Pre-induction treatment to allow cells to express arsenic-resistant proteins prior to arsenic exposure did not impact upon the number or morphology of colonies at lower arsenic concentrations, although higher IPTG pre-induction concentrations were deleterious. However, at high arsenic concentrations, of 60 mM and above, only pre-induced cells showed colony growth suggesting pre-induction is required for survival at high arsenic concentrations.

Strains containing the induced pArs_lac did not exhibit the same level of arsenate resistance as pEC20 cells but did show the same level of resistance as p2 + 3 cells, showing growth at 80 mM sodium arsenate after 3 days (Figure 5C). Thus, the lac operator only reduces the negative effects of EC20 over-expression but, while the cells grow more slowly, they are able to tolerate higher arsenic concentrations.

**Figure 6:**
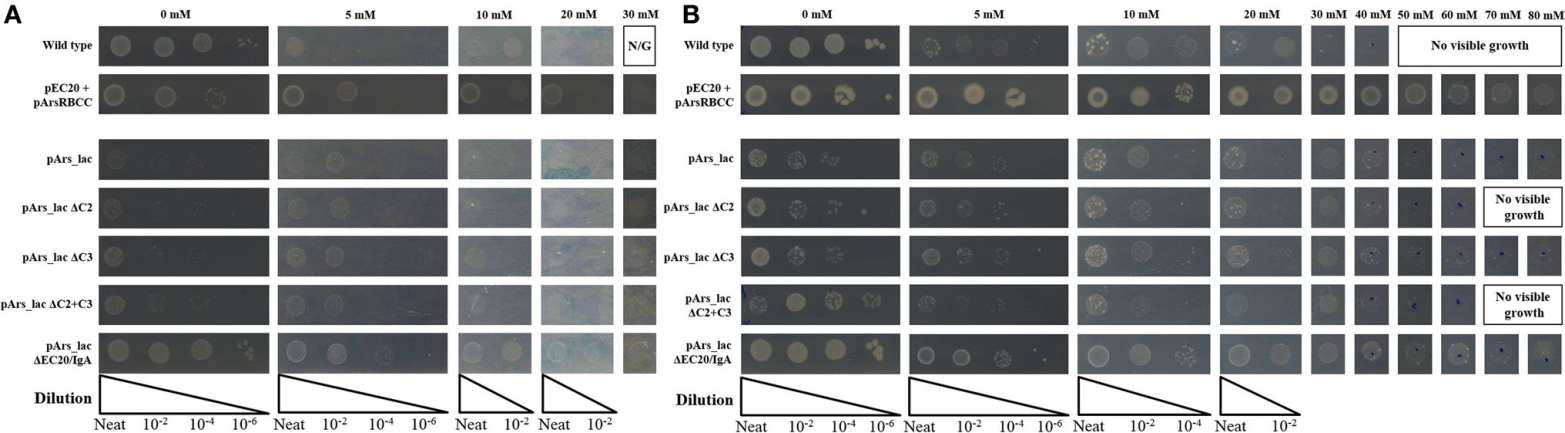
***E. coli* pArs_lac deletion variants gown on agar plates containing varying concentrations of sodium arsenate after (A) 16 h and (B) 3 days of incubation**.

Systematic deletion of modules from the pArs_lac construct produced changes in the level of arsenic resistance afforded to the cells. Tested here were the removal of ArsC2 (ΔC2) and ArsC3 (ΔC3), both individually and together (ΔC2 + ΔC3), and EC20/IgA fusion protein (ΔEC20/IgA). While all the deletion mutants show improved growth compared to the pArs_lac strain after 16 h (Figure 6A), when sodium arsenate is present the ΔC2, ΔC3, and ΔC2 + C3 mutants all showed similar growth to pArs_lac with a maximum tolerance of 30 mM sodium arsenate, with ΔEC20/IgA showing the highest colony density of all the strains tested here. After 3 days incubation ΔEC20/IgA displays greater apparent resistance to sodium arsenate than the pArs_lac and other mutant strains with the highest colony density at 80 mM (Figure 6B), likely due to a relaxation of growth restraints on the cells caused by EC20/IgA affecting cell division rather than an increased ability to deal with the sodium arsenate. While the ΔEC20/IgA mutation improves the growth of the pArs_lac strain it does not compensate for the resistance to arsenic afforded by EC20/IgA expression at lower levels, as p2 + 3 still shows the highest colony density at 80 mM sodium arsenate of the investigated variants.

### Quantification of Arsenic Removal

**Figure 7:**
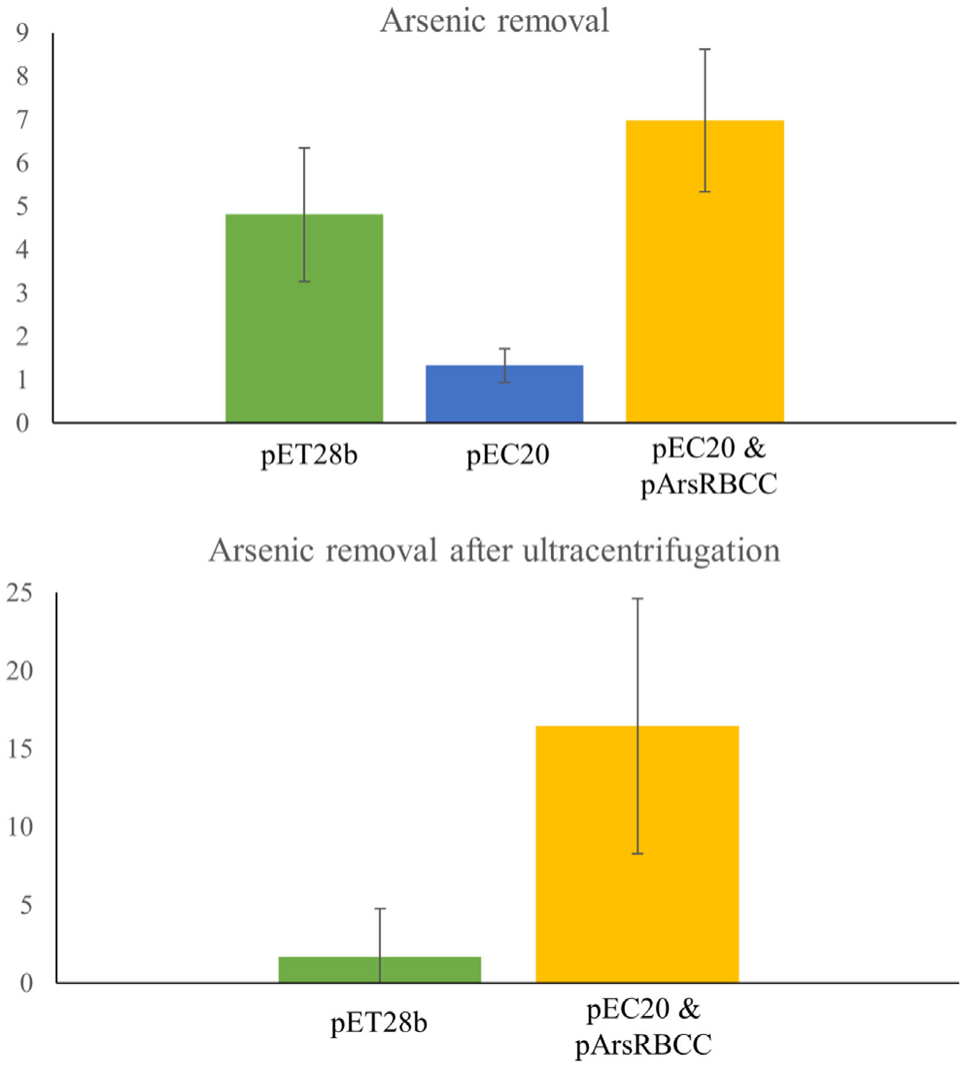
**ICP–OES analysis of arsenic removal. Top: arsenic removal analyzed after cells have been removed from sample by centrifugation and filtering. pET28b, pEC20, and pEC20 & pArsRBCC (p2 + 3) cells analyzed. Bottom: arsenic removal by pET28b and pEC20 & pArsRBCC (p2 + 3) cells analyzed after cells have been removed and sample ultracentrifuged**.

Quantification of nanoparticle synthesis was determined through measuring the sodium arsenate removal by cells using ICP–OES. The arsenic concentration was measured after the cells had been removed from the samples by centrifugation and filtering (Table 1). These results show that resistance to arsenic does not directly determine the amount of arsenic removed; pEC20 is able to survive sodium arsenate concentrations in excess of 80 mM and yet only removes around 1.3% of the arsenic, compared with pArsRBCC which has a maximum resistance of only 50 mM and which removes 6.9%. Ultracentrifugation of the samples increases the amount of arsenic removed but only for some of the strains. ICP–OES analysis of pET28b (as a control vector conferring kanamycin resistance) and pEC20 & pArsRBCC (p2 + 3) cells shows that while ultracentrifugation has little effect on the pET28b samples the amount of arsenic removed for the p2 + 3 strain is increased to 16% on average (Figure 7), and in individual cases this has been observed as being as high as 22% arsenic removal. This increase can be attributed to the arsenic nanofibers that this strain produces; ultracentrifugation may remove them where centrifugation at lower speeds has failed to do so.

**Table 1:** **ICP–OES analysis of arsenic removal by various strains, compared to the maximum arsenic resistance after incubation for 3 days**.

	Strain	% As removed	As resistance (mM)
Control plasmids	pUC19	4.2	20
	pHEβ	2.9	20
	pET28b	4.8	20
Single vector strains	pArsC1	3.0	50
	pEC20	1.3	>80
	pArsRBCC	6.9	50
Double vector strains	pArsC1 and pEC20	3.8	20
	pArsC1 and pArsRBCC	7.1	40
	pEc20 and pArsRBCC	6.7	80
pArs and pArs_lac	pArs	5.3	40–50
	pArs_lac	8.3	40–50
	pArs_lac + 0.1 mM IPTG	6.0	80

## Discussion

JM109 *E. coli* cells containing control plasmids possess a level of arsenic tolerance and synthesize arsenic nanoparticles. This may form part of their arsenic-resistance mechanism; by converting soluble, toxic arsenic into an insoluble form attached to the cell it is no longer able to enter the cell and cause damage. The current proposed mechanism for the action of the *E. coli* arsenic-resistance operon, ArsRDABC, involves the uptake of As(V) by the cell, reduction of As(V) to As(III) by ArsC, and the efflux of As(III) *via* the efflux pump/ATPase pair ArsAB (Saltikov and Olson, 2002). Further processing this arsenic into the insoluble, surface-bound As(0) detected in this study would increase arsenic resistance. Accumulation of nanoparticles at the cell surface, as observed with other metal-resistant bacteria (Capeness et al., 2015), is a component of the bacterial metal ion resistance mechanism. With our engineered strains of *E. coli* containing pArs_lac and pEC20 & pArsRBCC (p2 + 3) the cells take this mechanism to an extreme, producing very large As(0) structures, stabilizing large amounts of As and removing it from the solution.

The phytochelatin analog EC20, when expressed at the cell surface as EC20/IgA, increases the arsenic resistance of *E. coli*, so that after 3 days of incubation there is no difference between cells grown on plates containing no arsenic and those grown in 30 mM sodium arsenate (Figure 1A). The resistance mechanism employed here may involve the binding of As(V) to EC20/IgA, thereby lowering the effective arsenic concentration and giving the cells more time in which to process As(V) into As(III), and on to As(0). pArsC1 and pArsRBCC also increase resistance to sodium arsenate, but to a lesser extent (Figure 1). While all three plasmids individually increase sodium arsenate resistance when they are paired together they prove somewhat detrimental to the cells with all three possible combinations slightly reducing growth levels in the absence of sodium arsenate (Figure 1). This is most likely due to the increase in the protein expression load, with the cells overexpressing both the proteins of interest and two different antibiotic resistance proteins. In the presence of sodium arsenate, the resistance offered by the vectors outweighs the negative effects of the increased load, with pArsC1 & pArsRBCC (p1 + 3) slightly increasing and pEC20 & pArsRBCC (p2 + 3) greatly increasing arsenate resistance compared to the wild-type (Figure 1B), although pEC20 by itself still confers the greatest level of resistance.

Arsenic resistance, however, does not directly correlate with nanoparticle production; while pEC20 cells are able to survive high sodium arsenate concentrations the cells do not produce the large arsenic structures nor the fibers seen with pEC20 & pArsRBCC (p2 + 3) cells (Figure 3), and therefore the presence of the proteins encoded on pArsRBCC is a requirement for particle production. Similarly resistance does not directly influence the strains’ ability to remove arsenic from a solution (Table 1); there is a balance between the beneficial effects of the introduced proteins on arsenic resistance and the increased cost to the cells in producing them, and there are also the interactions of the various proteins influencing arsenic resistance and nanoparticle formation. The possible mechanism for the production of the large As(0) structures is that EC20/IgA may be acting as a “holding area” for excess arsenic, allowing the cells to survive in very high sodium arsenate concentrations; when present, the overexpressed genes of pArsRBCC then act to rapidly convert large amounts of As(V) into As(III) and export it from the cell, with further conversion to As(0) and its attachment to the cell surface. This rapid processing allows much larger structures to form compared to the control cells over the same amount of time. As analogs of these proteins are found in the genome of *E. coli* it may be that if allowed to continue for a sufficient length of time pEC20 cells would also show the larger surface-bound structure, with EC20/IgA preventing damage to the cell in the meantime. EC20/IgA may also play a role in promoting the formation of the large surface-bound As(0) structures and/or nanofibers by p2 + 3 cells, acting as nucleation points for the exported As(III) and facilitating its conversion to As(0).

In creating the modular construct, it was found that it had a detrimental effect on cell growth (Figure 5A). This is likely mostly due to the over-expression of EC20/IgA; as it is located at the cell membrane large amounts of the protein could be interfering with cell division, causing the elongated morphology seen in Figure 5A. This occurs even though the Ars operon repressor ArsR was included in the construct to allow for expression only when arsenic is present. Putting the lac operator sequence into the promoter to supplement ArsR greatly improves the growth of the cells, having around the same growth as the control cells after 3 days when no IPTG is present, and still allowing the cells to survive concentrations of arsenate up to 80 mM when they are induced with 0.1 mM IPTG (Figure 5C).

Modularizing the components of the arsenic resistance pathway has allowed us to investigate the role of these modules in arsenic resistance. The systematic module deletions demonstrated the detrimental effect of protein over-expression from the whole construct, as when ArsC2, ArsC3 (singly and together), and Ec20/IgA are deleted there is an improvement in cell growth (Figure 6). These deletion variants also demonstrated that using the native *E. coli* ArsR repressor and Ars operon promoter sequences have a significant effect on arsenic resistance. The ΔEC20/IgA mutation in effect gives the cells a similar genotype to the pArsRBCC strain, but while pArsRBCC uses the *D. alaskensis* ArsR and Ars promoter sequences with a maximum arsenate resistance of 50 mM (Figure 6), pArs_lac_ΔEC20/IgA is ameliorated for use in *E. coli*, and showed growth at 80 mM sodium arsenate (Figure 6). In addition, the data show ArsC3 is more important than ArsC2 in arsenic resistance, as ΔC3 cells show less resistance to sodium arsenate than the ΔC2 strain (Figure 6). Taken together, these data will allow us to optimize the construct to maximize arsenic resistance while minimizing the detrimental effects of protein over-expression.

The modularized pArs_lac vector provides easy adaptability. If, for example, contamination to be remediated is known to contain only As(III), and not As(V), then the reductase genes could be removed to reduce the protein expression load on the cells. Similarly, if a different metal was to be investigated, the arsenic-specific components could be switched for others specific to the new contaminant. While pArs_lac (with 0.1 mM IPTG) displays lower growth than p2 + 3 at 80 mM sodium arsenate (Figure 5C), pArs_lac offers the greatest degree of flexibility owing to its modular nature; as the most desired aspect of the construct is utility rather than maximum arsenate resistance it is pArs_lac that will be the focus of future work. The modularity also has the advantage of that in the future our construct can be further adapted to dealing with other metals and metalloids.

Using a biological approach to arsenic remediation offers a number of advantages over other chemical or mechanical methods. By using living organisms, it is very straightforward to replace or increase the amount of bioremediating material as the cells can be grown in normal growth media, reducing material costs; in addition, the nature of living organisms makes them almost infinitely adaptable, something we can further use to our advantage by applying the engineering principals of synthetic biology in our modification of the bacteria. Our work has also created new tools for the bioremediation of arsenic by utilizing previously studied proteins in new ways. Phytochelatin, for example, has been investigated in remediation or nanoparticle production contexts due to its affinity for many different heavy metals and metalloids (Park et al., 2010; Singh et al., 2010); in many of these cases the protein is expressed inside the cell, but we have hybridized it with the membrane-spanning protein IgA, allowing it to be transported to the outside of the cell. When combined synergistically with other proteins in our work, this results in *E. coli* strains that tolerate high levels of arsenic and which are able to convert arsenic ions into large, stable As(0) structures. Having the arsenic on the outside of the cell rather than internally may mean that the cell is able to handle higher amounts of arsenic, as well as survive longer, potentially allowing more arsenic to be removed using our method.

We have demonstrated that our engineered arsenic-resistant *E. coli* strain is able to survive high levels of sodium arsenate, up to 80 mM, and can remove significant amounts of the arsenic by converting it into stable, insoluble metallic As(0). Our modularized plasmid allows the activity of the strain to be tuned for purpose, being able to add or remove modules as required, providing a flexible way of removing arsenic from a number of waste sources.

## Author Contributions

ME and LH designed experiments. ME performed experiments. ME and LH wrote the manuscript.

## Conflict of Interest Statement

The authors declare that the research was conducted in the absence of any commercial or financial relationships that could be construed as a potential conflict of interest.

## Footnotes

^1^http://www.arsenicbiosensor.org/index-2.html

^2^All accession numbers are from the NCBI database
