# Supplementary material for: Construction of a Modular Arsenic-Resistance Operon in E. coli and the Production of Arsenic Nanoparticles
Source: Front Bioeng Biotechnol. 2015 Oct 20;3:160. doi: 10.3389/fbioe.2015.00160 (PMC4611968; doi:10.3389/fbioe.2015.00160)
Supplement: Supplementary file 1 [file data_sheet_1.docx]

**Table S1: Plasmid origins**

| **Vector** | **Origin** | **Antibiotic resistance** |
| --- | --- | --- |
| pArsC1 | pUC19 | Ampicillin |
| pEC20 | pAK100 | Chloramphenicol |
| pArsRBCC | pUC57-Kan | Kanamycin |
| pArs | pUC57-Kan | Kanamycin |

**Figure S1: Sequence of genes used in constructs ameliorated for expression in *E. coli***

**ArsR**

atgagcttcctgctgccgatccagctgttcaagatcctggccgacgagacccgcctgggcattgtgctgctgctgagcgagctgggcgagttatgcgtgtgcgacctgtgcacagccctggaccagagccagccgaagatcagccgccacctggccttactgcgcgagagtggtctgctgctggaccgcaagcagggcaagtgggtgcactaccgcctgagccctcacattcctgcctgggccgccaagatcatcgacgaggcctggcgctgcgagcaggagaaggtgcaggccatcgtgcgcaatctggcccgccaaaactgcagcggcgacagcaagaacatctgcagctaa

**ArsB**

atgggatcccacgaaagcgttgttaagaagctgagcttcctggaccgttacctgaccctgtggatcttcctggcaatgtttgtgggtgttggcggtggctacctgtacccggacgtgaaaaatgtgatcaatagttttcaggttgggacgaccaacattccgattgccatcggcctgattctgatgatgtacccgccgctggccaaagtgaagtacgagcagctgggccaggtttttcgcaacttcaaggtgctggcactgagcctggttcagaactggatcattggcccgatcctgatgttcggcctggccattacctttctgagtggttaccacgagtacatggtgggcttaattctgattggtctggcccgttgcattgcaatggtgatcgtgtggaacgatctggccaagggcgatacagagtattgcgccggtttagttgcattcaacagcatctttcaagtgatattcttcagtgtgtatgcctacctgttcatcaccgtgctgccgggttggttcggcctgaaaggtgtggttgtggacattagcatcggccagattgcagagagcgtgttcatctacttaggtattccgttcatcgcaggcatgttaacccgtttcatgggcctgaagctgaagggtcaggaatggtacgaaaacacctttattccgcgcatcagtccgctgaccctggttttcctgttattcacaatcctggttatgttcagcttaaaaggcgaaaaggtgattcagttaccttttgacgtgatccgtatcgccatcccgctgacaatctattttttagtgatgtttctggttagtttttacttaagctataaagcagatgcaacctacgagcaggccaccacattaagtttcaccgccgccagcaataactttgagttagccattgcagttgcaatcgccgtttttggcattaacagcggtgaggccttcgccgccgttattggtcctctggtggaggttccggtgctgattgccctggtgaacgtggccctgcacttcaaacgcaagtatttcccgaaagcagttgagaccattgcaggtgtgtgccacgtgaaatgtgattaagatatc

**ArsC1**

atgccgatgaagaagaccatcctgttcctgtgcaccggcaacagctgccgtagccagatggcagagggctggacccgcaa
gctgaagggcgacgagttcgaggtgcgcagtgcaggtgtggagacccacggcctgaatccgctggcagtggaggtgatgg
ccgaggcaggcgtggacatcagtggccaccgcagcaagctggtgagcgagctggacgtggacacctttgactacgtggtg
accgtgtgcgatcacgccagcgagcactgcccgttctttccggcagagagtcgccgcattcacgccggtttccctgatcc
gcctgcattagcccgcaccgcagccacacacgaggaagcactggagcactatcgtcgcgtgcgcgacgccatccgcgact
ttgttcagagcctgccggccccgctgttataatccgtgtccggaattccggccgcagaatctag

**ArsC2**

atgaagcttgccaaggccgacctgaaacagctgggtggcgacgagttccaggtggaaagcgccggcttcaagccgaccgagatcaacccgatgctggtgaaggtgatgcaagaagaggggatggacctggcaagcaacctgacccaggccgccttcgaaatattcaagaagggccgcaccttcacctacgtgatcaccgtgtgcgacaagagcgtggacgacaactgcccgatcttccagggcatgatgtaccgcctgcacctgcatttcgcagcaccggccaaggtggacgccaccaaagaggagaagctgaccgagttccgcatcatccgcgaccagatcaaggccatcgcccaggagttcatcgactgggtgcgcgccggcgacaagaagaagctgggcgacctgtgggacaccaaggacatccgcaagggcgagctgcactaa

**ArsC3**

atggaacatcctgttcctgtgcacaggtaacagctgtcgcagtcaaatggcagagggttgggcacgccatctgaaggcagacatattcaatgcctacagcgccggtgtgaagaaacacggtctgaaccctctggcagtgaaggcaatggcagaagccggtgttgacattagcaaacaggagagcaagaccattgacgagctgcctcaggacgttgagttcgattacgttgttacagtgtgcaataacgcccacgaacaatgccctttctttccggccaagagcaagatcgtgcatgttggtttcgacgacccgcctgcactggcaaaaaacgttaacaacgaagaagaggcgctgcaaatctaccgccgcatccgtgacgagattaagattttcgttagtaacctgcctgactgcgtggttaccaacagcgtgtaa

**Ec20/IgA**

atgagatctaagtacctgctgcctaccgcagcagccggcttattattactggcagcacagccggcgatggcggagtttgagtgtgaatgcgaatgtgagtgcgagtgtgaatgtgagtgtgagtgcgagtgcgaatgcgaatgcgaatgtgaatgcgagtgcgagtgcgaatgcgaatgcgagtgtgagtgtgagtgctctagaggtgcacctgtgccgtaccctgatccgctggagcctattgacaacagtgccgccatcagcatggccaaccctcgtccgcctacccctcgtgcagccgcagcagtgttcagcttagatgactacgacgccaaggataacagcgagagtagcattggcaacctggcacgcgtgattccgcgtatgggtcgcgaactgatcaatgattatgaggagattcctctggaggaactggaagacgaagccgaagaggagcgccgtcaggcaacacaatttcagccgaagagccgtaaccgtcgtgccatcagcagcgagcctagtagtgatgaggatgccagtgaaagcgtgagtaccagcgataaacaccctcaggataacaccgagctgcacgagaaagtggagaccgcaggtttacagcctcgcgcagcacaacctcgtacacaggcagcagcccaggcagacgcagtgagtaccaacaccaacagcgccttaagcgacgcaatggcaagcacccagagcatcctgctggacaccggcgcatacttaacccgtcatatcgcacagaagagtcgcgcagacgccgaaaagaacagtgtttggatgagtaacaccggttatggtcgcgattacgcaagcgcccaataccgccgcttcagtagcaaacgcacccagacacagatcggcattgaccgcagcctgagcgaaaacatgcagattggcggcgttctgacctatagcgacagtcagcacaccttcgatcaggcaggcggcaagaacacattcgtgcaggccaatctgtacggcaaatactatttaaacgacgcatggtatgttgcaggcgacatcggcgcaggcagtctgcgcagccgtttacagacccagcagaaggcaaactttaaccgcaccagtattcagacaggcctgaccctgggcaataccctgaaaatcaaccagtttgagattgtgccgagcgccggcattcgctatagccgtctgagcagcgcagactataagctgggtgacgacagcgttaaagtgagcagcatggccgtgaaaaccctgacagcaggtctggactttgcctatcgcttcaaagtgggcaacctgaccgttaagcctctgctgagcgcagcctacttcgcaaactacggcaagggcggcgtgaacgttggtggcaagagttttgcctacaaggcagacaatcaacaacagtacagtgccggtgtggccctgctgtaccgtaacgttaccctgaatgttaacggcagtatcacaaagggcaagcagctggaaaaacagaagagtggccagatcaaaatccaaattcgtttctaa

**Figure S2: Protein expression in all strains, soluble fraction**


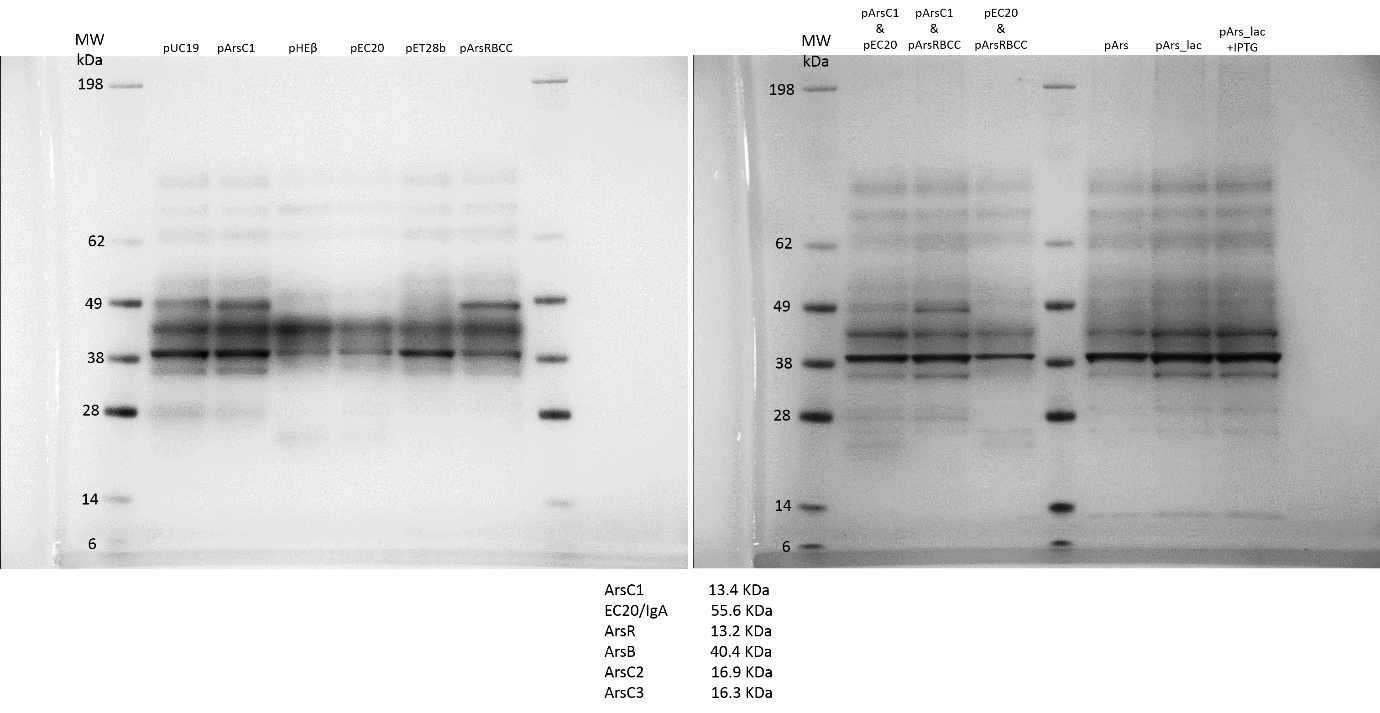


Protein expression levels in strains containing single or double plasmids (pArsC1, pEC20 and pArsRBCC; pArsC1 & pEC20, pArsC1 & pArsRBCC and pEC20 & pArsRBCC) are not expressed at high levels. Protein expression in the pArs strains with plasmids based on pUC57-Kan (pArs, pArs_lac and pArs_lac + 0.1 mM IPTG) is high enough to be observed on the gel. ArsR is observable at 13.2 KDa, with slightly high expression in the pArs_lac cells, and ArsB may be visible at 40.4 KDa as a slight thickening of the band here compared to all the other strains. EC20/IgA is not readily visible, separate analysis of the membrane fraction may be necessary.

**Figure S3: Primer sequences**

“Lac operon forward”, ggataacaattccaatcaggagcgcCATATGgag

“Lac operon reverse”, gctcacaattccttgcaggtagtgtctctcttc
